# Supplementary material for: Identification of the Factor That Leads Human Mesenchymal Stem Cell Lines into Decellularized Bone
Source: Bioengineering (Basel). 2022 Sep 21;9(10):490. doi: 10.3390/bioengineering9100490 (PMC9598111; doi:10.3390/bioengineering9100490)
Supplement: Supplementary file 1 [file bioengineering-09-00490-s001.zip › bioengineering-1862916-supplementary.pdf]

Supplementary Figure S1

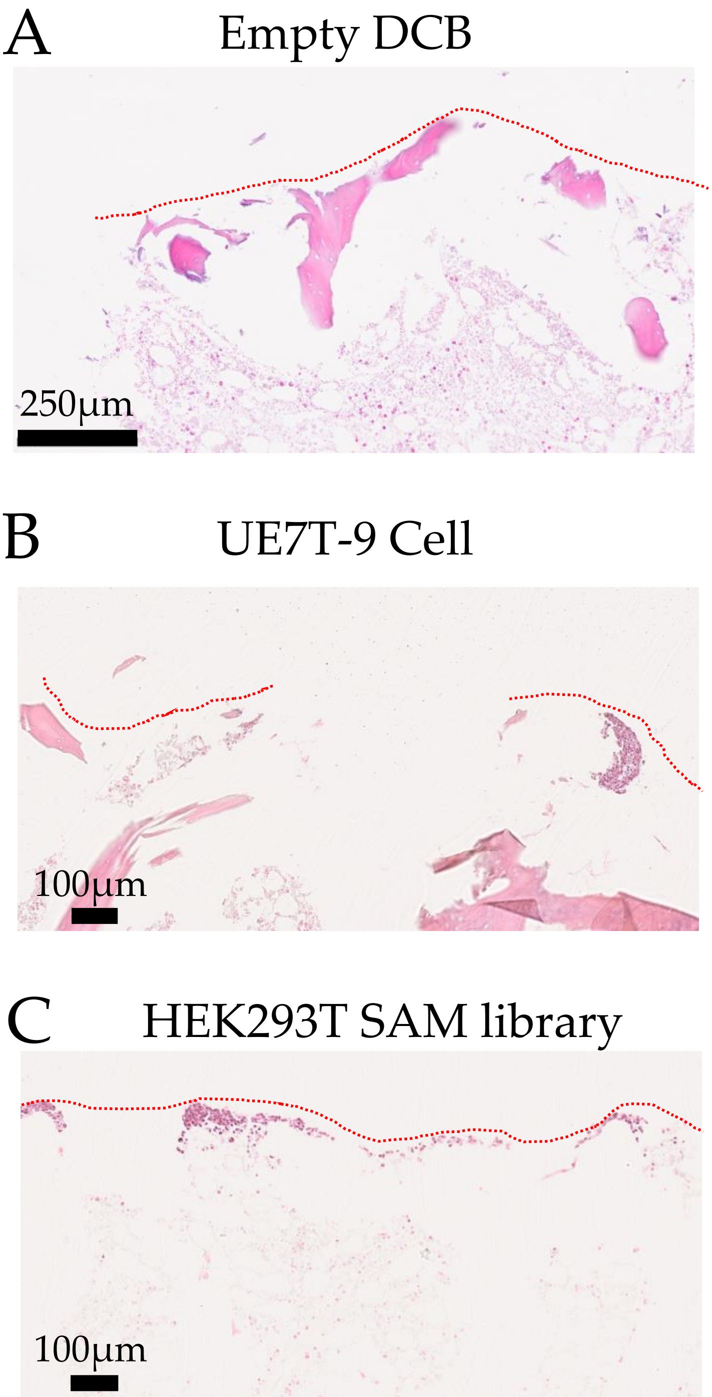

Supplementary Figure S2

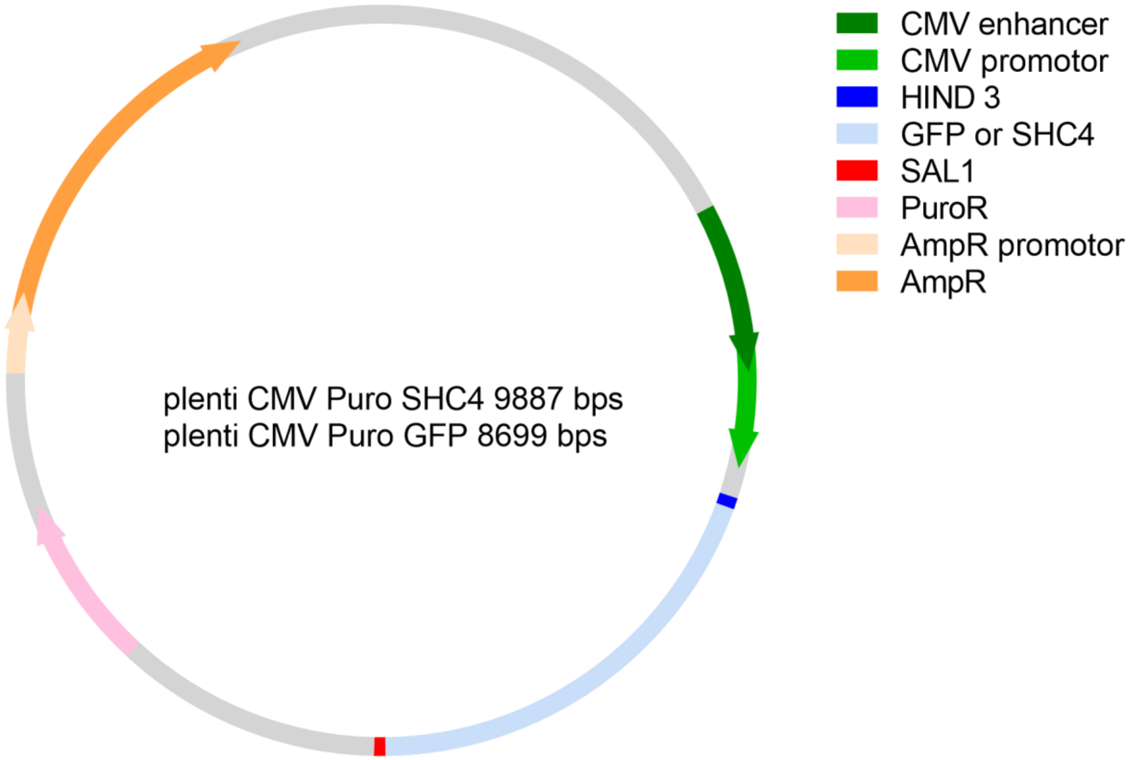

Supplementary Figure S3

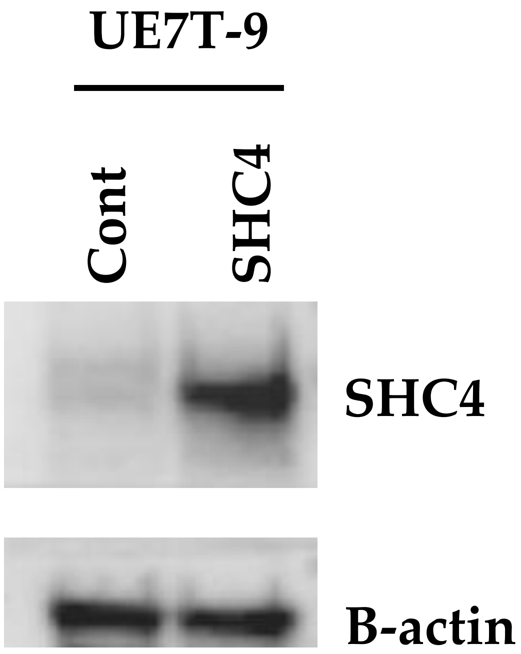

Supplementary Figure S4

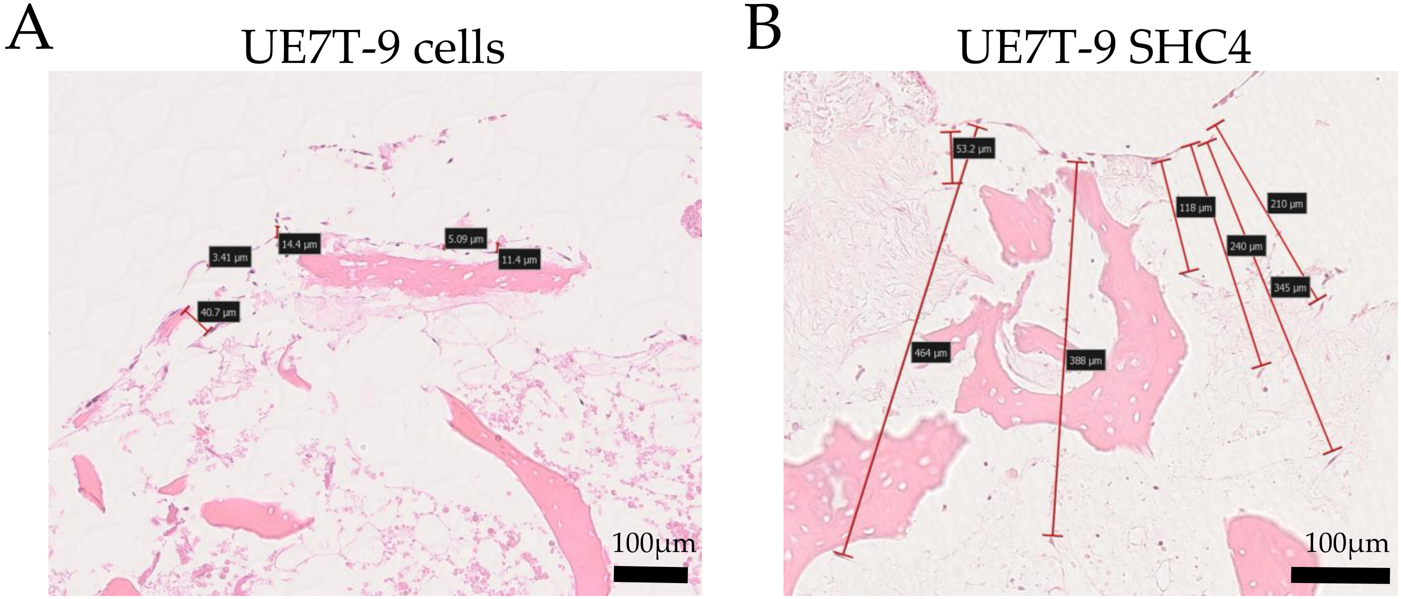

Supplementary Figure S5

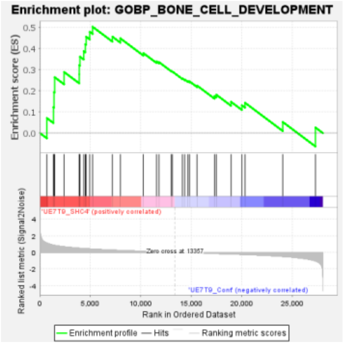

NOM p-value = 0.0
FDR q-value = 0.10485437

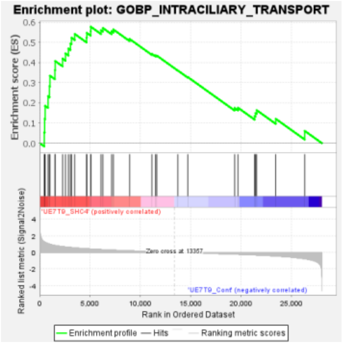

NOM p-value = 0.0
FDR q-value = 0.0945674

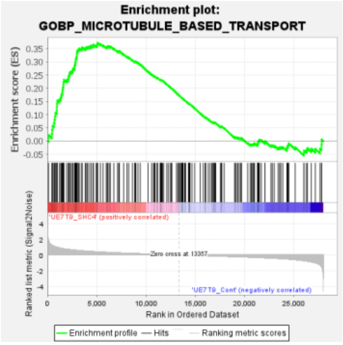

NOM p-value = 0.0
FDR q-value = 0.11875

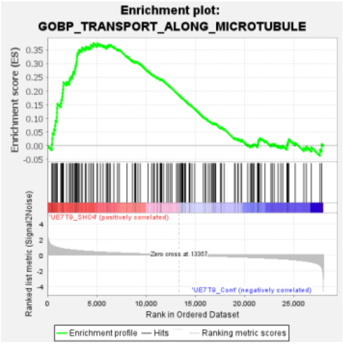

NOM p-value = 0.0
FDR q-value = 0.10810811

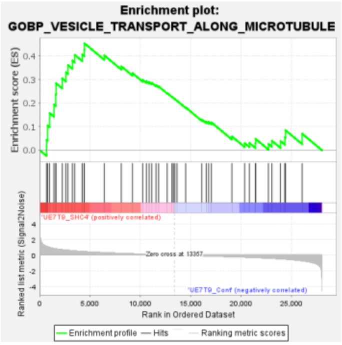

NOM p-value = 0.0
FDR q-value = 0.086065575

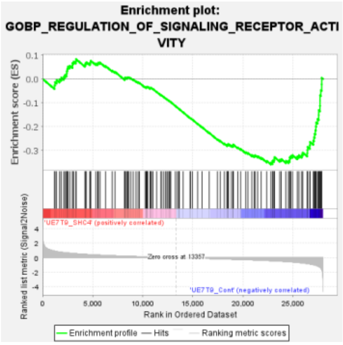

NOM p-value = 0.0
FDR q-value = 0.0945674

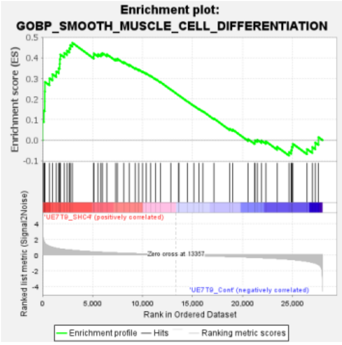

NOM p-value = 0.0
FDR q-value = 0.08634538

Supplementary Table S1

GOID	Description	GeneRatio	BgRatio	pvalue	padj	Count
GO:0097529	myeloid leukocyte migration	18/282	145/14579	3.85E-10	1.29E-06	18
GO:0010469	regulation of signaling receptor activity	28/282	397/14579	3.51E-09	3.34E-06	28
GO:1990266	neutrophil migration	13/282	80/14579	3.99E-09	3.34E-06	13
GO:0097530	granulocyte migration	14/282	97/14579	4.95E-09	3.34E-06	14
GO:0060326	cell chemotaxis	20/282	215/14579	6.86E-09	3.34E-06	20
GO:0050921	positive regulation of chemotaxis	15/282	117/14579	7.38E-09	3.34E-06	15
GO:0030593	neutrophil chemotaxis	12/282	70/14579	8.51E-09	3.34E-06	12
GO:0071621	granulocyte chemotaxis	13/282	85/14579	8.58E-09	3.34E-06	13
GO:0050920	regulation of chemotaxis	18/282	176/14579	8.98E-09	3.34E-06	18
GO:0032103	positive regulation of response to external stimulus	20/282	241/14579	4.74E-08	1.59E-05	20
GO:0071622	regulation of granulocyte chemotaxis	9/282	41/14579	6.80E-08	2.07E-05	9
GO:0030595	leukocyte chemotaxis	16/282	161/14579	9.16E-08	2.55E-05	16
GO:0030335	positive regulation of cell migration	26/282	426/14579	2.36E-07	6.08E-05	26
GO:0002688	regulation of leukocyte chemotaxis	12/282	95/14579	2.84E-07	6.80E-05	12
GO:0008015	blood circulation	26/282	436/14579	3.70E-07	7.84E-05	26
GO:0003013	circulatory system process	26/282	438/14579	4.04E-07	7.84E-05	26
GO:0040017	positive regulation of locomotion	27/282	468/14579	4.20E-07	7.84E-05	27
GO:2000147	positive regulation of cell motility	26/282	439/14579	4.22E-07	7.84E-05	26
GO:0050900	leukocyte migration	22/282	335/14579	6.12E-07	0.000108	22

Supplementary Table S2

NAME	SYMBOL	TITLE	RANK IN GENE LIST	RANK METRIC SCORE	RUNNING ES	CORE ENRICHMENT
row_0	MIR27B	na	80	2.006150484	0.0784945	Yes
row_1	JCAD	na	523	1.201081514	0.11138476	Yes
row_2	STAT5A	na	526	1.199920297	0.15997483	Yes
row_3	THBS1	na	537	1.194373012	0.20805363	Yes
row_4	CD40	na	1440	0.842455328	0.20993747	Yes
row_5	FGF18	na	2077	0.720758319	0.21640572	Yes
row_6	PDGFB	na	2671	0.637656808	0.22104277	Yes
row_7	PRKD1	na	2750	0.628588557	0.24374309	Yes
row_8	HSPB1	na	2992	0.600527883	0.25947192	Yes
row_9	MAP3K3	na	3473	0.543644011	0.26434046	Yes
row_10	MIR10B	na	3793	0.509561718	0.27358878	Yes
row_11	FGFR1	na	4217	0.470974565	0.2775502	Yes
row_12	ATP5F1A	na	4856	0.420867354	0.27178508	Yes
row_13	TMSB4X	na	4890	0.419208437	0.28760466	Yes
row_14	RHOJ	na	5106	0.401287049	0.29618397	Yes
row_15	HDAC7	na	5159	0.397529751	0.3104444	Yes
row_16	PLCG1	na	6913	0.288059354	0.2593893	No
row_17	AKT3	na	7009	0.282970339	0.267465	No
row_18	SP1	na	7082	0.278080672	0.27616554	No
row_19	PLK2	na	7236	0.268707573	0.2815871	No
row_20	CIB1	na	8085	0.229117215	0.26053014	No
row_21	HMOX1	na	8475	0.212350413	0.25522014	No
row_22	ETS1	na	8488	0.211902097	0.26338416	No
row_23	HDAC9	na	8731	0.200153291	0.2628404	No
row_24	FOXC2	na	9806	0.151230082	0.23053662	No
row_25	MIRLET7F1	na	9829	0.150917947	0.23586962	No
row_26	VEGFC	na	11753	0.069871381	0.16988206	No
row_27	NFE2L2	na	12017	0.059476808	0.16288173	No
row_28	NRP1	na	12410	0.045382153	0.1506931	No
row_29	MIR221	na	13591	-0.009490869	0.108847655	No
row_30	HIF1A	na	13602	-0.009789514	0.10888678	No
row_31	AKT1	na	13806	-0.020414256	0.10244961	No
row_32	NOS3	na	14773	-0.048365697	0.06983942	No
row_33	MAPK14	na	14806	-0.049554404	0.07070383	No
row_34	TGFB1	na	14889	-0.052395739	0.06989403	No
row_35	P2RX4	na	15316	-0.067835391	0.05739917	No
row_36	VEGFA	na	15646	-0.080824926	0.048902556	No
row_37	PRKCA	na	16392	-0.105386086	0.026514018	No
row_38	ANGPT1	na	17236	-0.141899601	0.002098987	No
row_39	HMGB1	na	18763	-0.20597811	-0.04416089	No
row_40	ATP5F1B	na	19150	-0.221499115	-0.04899253	No
row_41	NUS1	na	19914	-0.252773672	-0.06604809	No
row_42	ABL1	na	20104	-0.260890752	-0.06223192	No
row_43	PDPK1	na	21219	-0.316465229	-0.08926627	No
row_44	MIR210	na	21709	-0.343527526	-0.09283537	No
row_45	SIRT1	na	21890	-0.353086591	-0.08495819	No
row_46	MAP2K3	na	22056	-0.362186193	-0.07617515	No
row_47	ADAM17	na	22381	-0.383765727	-0.07220734	No
row_48	GATA2	na	22406	-0.385374069	-0.05743777	No
row_49	PTGS2	na	23283	-0.441656172	-0.07087747	No
row_50	PIK3C2A	na	24041	-0.499452233	-0.07771449	No
row_51	ANGPT4	na	25187	-0.616642952	-0.093684845	No
row_52	PRKD2	na	25286	-0.628580928	-0.071700595	No
row_53	FGF2	na	25834	-0.701947331	-0.06281004	No
row_54	ANXA1	na	25931	-0.716482401	-0.03718945	No
row_55	KDR	na	27411	-1.101939321	-0.0454324	No
row_56	SRPX2	na	27877	-1.637428403	0.004330372	No
